# Supplementary material for: Identifying a developmental transition in honey bees using gene expression data
Source: PLoS Comput Biol. 2023 Sep 21;19(9):e1010704. doi: 10.1371/journal.pcbi.1010704 (PMC10547183; doi:10.1371/journal.pcbi.1010704)
Supplement: Attachment — Submitted filename: bistability_reviewer_response_v3.pdf [file pcbi.1010704.s002.pdf]

We are grateful for the thoughtful and constructive feedback from all three reviewers. Below, we respond in detail to all comments, with reviewer comments in *italics* and our changes to the manuscript highlighted in green.

Reviewer #1

Daniels et al. presents a computational method for detecting a bifurcation (of the sense of nonlinear dynamical systems) in a gene network consisting of mutually inhibiting genes (loosely speaking, see below) that governs the behavior in bees. The bifurcation is proposed to underly the developmental swicth between performing tasks in the nest and leaving the nest for foraging. The algorithm is designed to detect the appearance of a bimodal distribution of gene expression values of key regulatory genes involved in this switch in a population of bees, as a manifestation of a symmetry-breaking (pitchfork) bifurcation. The principle is essentially the fit of the distribution to a model of bimodal distribution. Specifically (the novelty), the author use a model derived from dynamical systems theory and Landau theory to compute the equilibrium state in a stochastic system in a bistable regime. They show that their model fits the data (the distribution of expression of key regulatory genes in bees of a population of N=16 bees) better than simply assuming two Gaussians (gaussian mixture model) to model the bimodal data, hence detect the bifurcation.

OVERALL:
Superficially, this work looks like a heuristic, albeit well-informed, approach of fitting distributions. But this work is more than that. It is of great originality and connects a bottom-up theory based approach (where theory encompasses dynamical systems theory and Landau theory) with the statistical approach of fitting the data to a model. And it does so in a logically coherent, hence satisfactory manner, which is rare in the current landscape of theory-free, big-data-driven bioinformatics.

While one could further dissect the logical structure of this confluence of two distinct methodological ideologies for its actual epistemological merit, which would require the skill of a historian or philosopher of science, I found, as a practitioner, this work by Daniels and coauthors most elegant and interesting, one of the most profound yet simple and enjoyable read for a long time in computational biology. While one may criticise the small number of bees analyzed, the logical and theoretical narrative is compelling, such that one could view the presented validation using real-world data as a first step, a demonstration example.

It thus appears that, modulo some simplifying assumptions in the theory and modeling that one could discuss, this work is of high scientific merit. In these days of floods of bioinformatics tools based on ad hoc heuristics, the approach presented here will likely stand out and also be most useful: the core idea is simple, but solidly rooted in theory of dynamical systems considerations, and the purpose of the method is clear from the beginning, addressing an unmet need that is well relatable by a life science audience increasingly aware of the dynamical systems principles that underlies developmental switches.

For this reason, I expect this ms. to have impact, notably in the growing field of single-cell transcriptomics. This work is also overall (modulo points below) well written, with logical clarity throughout both the theoretical as well as the biological parts.

We thank the reviewer for their time and appreciate the enthusiasm regarding the style of our methodology and its potential applications.

MAJOR ISSUE:
Unfortunately, I could not see an indication of any effort to share the code.

We have now released on GitHub all code needed to reproduce our analysis on our dataset and apply it to other datasets: https://github.com/Collective-Logic-Lab/landau

OTHER ISSUES THAT, IF ADDRESSED MAY HELP FURTHER IMPROVE THE MANUSCRIPT:

(0) While the authors adequately present prior work in this area of detecting bifurcations, it would be useful to make a clear distinction upfront a bit more explicitly: Most prior work has been in the tradition of "Early Warning Signals" of critical transitions and seek to PREDICT an IMMINENT bifurcation (pitchfork, or saddle node) BEFORE it happens. The algorithms have a validity that is strictly limited to before the bifurcation point but not after (when bimodality is present). By contrast, the author's new approach, similar to that of one cited reference (19), seeks to DETECT the earliest indication of bimodality AFTER the bifurcation has happened.

We agree that connecting to previous literature becomes more coherent when we make a clear distinction between methods that detect increasing fluctuations before a transition versus bimodality after a transition. We now state in the introduction that bifurcations "can be identified by monitoring for increased correlated fluctuations in gene expression before the transition [17,18] or by testing for evidence of bimodality in gene expression after the transition [19,20]". We also emphasize in the results of Section 3 that the transition index method we compare to looks for critical signatures "*before* the split to bimodality".

(1) A main question that should be presented upfront with clarity to those readers familiar with the theory of dynamical systems and concepts of bifurcations, but not the specific examples of the bee behavior, is the following: Wha interactions drives the dynamics, and what is a dynamical system defined here? This is important because the detection of bifurcation based on a statistical example of replicate instances of the very same dynamical system obliges us to define what are the interactions and what is the dynamical system whose state variables exert or are controlled by the interactions. Instead the authors almost nonchalantly switch between three levels of interactions – and hence three levels of systems, without explicitly declaring them, which can be confusing: (a) Interactions between genes (e.g. lines 75, or lines 167, 186 ad more): Authors talk about "gene network". In the field of dynamical systems applied to biology it is a long tradition to use this term for INTRACELLUALR gene regulatory networks. In this case

the level of the system is the CELL each with its own (identical) internal GRN, and we examine ensemble of cells
(b) Interactions between cells, tissues (e.g. lines 45 "endocrine"; line 175+ "blood levels...") : Here an INTRA-INDIVIDUAL, INTER-CELLULAR (hormonal) network is suggested. In this case the system is the organism and we examine ensemble of organism (bees)
(c) Interaction between individual bees (e.g. line 51 "interaction with other foraging bees, ... ; line 181+). It looks like there is some sort of quorum sensing in the bee colony. In this case the dynamical system is the colony.

This distinction is important for a proper formulation of the theory using the dynamical systems formalism. The method requires a stochastic ensemble of instances of systems that are in principle identical and independent (generating the distribution being measured and fitted with the model), and whose high-dimensional features (expression values of genes) that interact (and form the feedback loop) drives the dynamics; therefore one has to be clear in the system description. Strictly speaking (1) and (2) are OK, here. But: in (3) having interactions between the systems (bees) which are the members of the distribution being evaluated, is a different matter that may complicate the model! However, as seen in other systems, the existence of such interactions may not disrupt the bifurcation behavior (but possibly bias it, e.g. making it asymmetric). But for the formalism and model it is important to have a clean description.
In any case, this classification is important for sake of being precise; the authors cannot have it multiple ways, even if I don't think this issue will affect the strength of results. They shold be clear about the very system they examine upfront and not muddle the levels of system

This is a subtle point that we had not previously fully appreciated, and we thank the reviewer for bringing it to our attention. We consider dynamics at all three levels that the reviewer mentions to be important to the phenomenology of the transition. Within individual bees, we expect that interactions among genes create bistability, since bees transition from in-hive to foraging activities even when they are isolated from others. Interactions of bees with their local environment, including other bees, then bias the bistability toward one of the two states, leading to transitioning earlier or later to the foraging state. While our phenomenological method cannot easily disentangle these levels of interactions, we can make progress by assuming that interactions between bees are small enough that they do not significantly alter the form of the bistability, only biasing it. This is somewhat analogous in cellular differentiation to looking for critical transitions in single cell data when those single cells are interacting in a tissue—in our case the distinction is between single bees and bees interacting in a colony.

To clarify what we intend interactions to represent in the model, we add the following paragraph in the Methods section:

In our simple dynamical model, we think of interactions as capturing dependencies between gene expression within an individual bee. Neglecting interactions between bees simplifies the interpretation of the resulting probability distributions, as each sampled bee represents an independent sample. Yet interactions between bees are known to bias the

transition. The simplest interpretation we could hope for is that these interactions are small, so that the distribution we observe experimentally is a good approximation for the case of non-interacting bees. This interpretation more easily explains the fact that bees transition from in-hive to foraging activities even when they are isolated from others. In the most difficult case, interactions between bees could be mostly responsible for the feedback that leads to bistability, with corresponding changes to gene expression within each bee that are driven by but do not cause the transition. Even in this case, our method provides a way to characterize the transition phenomenologically in terms of the degree of bistability and the genes that are associated with it.

(2) LANDAU model vs. GMM: In this comparison, it would be good to add two elements:
- The statistical significance for the claim, which is based only on visuals ("performs noticeably worse") of Fig. 3, that LANDAU model is better. There are simulations for N=100 and N=50,000 in Fig 3A and B, respectively. At least for Fig 3A, error bars should be shown or described (if too small). A statistical test of significance (KS?) may be useful.

We have now added error bars in Fig. 4A (the figure to which the reviewer was referring), which visually demonstrate that the Landau model identifies bistability significantly more often than the Gaussian mixture model in more difficult cases near the transition. In addition, we now quantify the significance of the difference between the two models in fitting the data with 50,000 samples:

> Comparing the best fit of the two distributions to 50,000 samples from the model close to the transition (Fig. 4B), the Landau distribution is not significantly different from the sampled data (KS statistic = 4.2 x 10^-3, *p*=0.33), while the Gaussian mixture distribution, with one more parameter than the Landau model, is significantly different (KS statistic = 0.017, p<10^-10).

- But even if the Landau model significantly better: This per se does not mean that it is better because of the reason claimed, that it is a "bottom" up theoretical model as opposed to the just phenomenology of the distribution. Could there be other "ad hoc" non-Gaussian distribution-based mixture models that could provide a better fit of the data than the GMM? (This could be discussed or evaluated on the simulated model)

We include the demonstration of the fit of the simulated data to the Landau distribution mainly as a way of visualizing what one would be missing by using the wrong form of distribution. It is also a simple "sanity check" for our mathematical analysis: We expect the distribution to be a good fit sufficiently near the transition because we show this analytically. (We could imagine other ad hoc distributions that could also do better than the GMM, but we see no good reason to use them: we still expect the Landau distribution to fit best sufficiently near a transition, and it remains relatively simple with fewer fit parameters than the GMM.)

(3). FIG. 5: The comparison with the previously critical reported transition index. It is interesting that here the latter works only with genes selected a priory to be involved in the transition. Note that several groups have used this or simialr approach as a tool to

actually IDENTIFY the genes involved in the transition (e.g. PMID 23230504, similar to ref 18 that the author cite). It must be stated for clarity that (a) This transition index method is actually inherently agnostic of choice of genes. But also that (b) that in this particular case -and in others- it seems that it works best with genes that play a role in he transition. A brief discussion of this interesting observation could be provided these relevant genes may be biased to fluctuate more in the pre-transition phase already along the dimension of the bifurcation.

We now clarify this point in the results in Section 3: "We note that the original formulation of this measure did not assume that the most important genes were known *a priori* [17], though this seems to be necessary to identify the transition when applied to our data."

We also include a new paragraph in the Discussion:

> An advantage to our approach is the ability to include a large number of genes that may or may not be related to a transition and then identify those individual genes that are most informative about the transition. Previous approaches to identifying transition states from transcriptomic data have also attempted this, though they have some limitations. One approach that analyzes critical fluctuations of gene expression across time has been used to identify genes involved in a transition [18] but requires detailed knowledge of protein interaction networks. Second, the transition index approach that we compare to in Section 3 has been shown, when applied to other datasets, to be robust to including large numbers of genes that may or may not be closely related to a given transition [17]. Yet this does not appear to be the case for our data, in which the transition is only detectable by the transition index measure once less informative genes have been filtered out (Figure 5).

Though detailing precisely why the transition index method does better when focused on individually informative genes is beyond the scope of this work, it is potentially related to the fact that bee-bee correlations remain always large (with correlation coefficients near 1) across all ages unless we focus on these informative genes.

(4). For the simulation model (Lines 421 ff, eq 19): It is not clear how the N-dimensional system (N=91) produces a bistable dynamics. Details may be in ref. 52, but that paper is inaccessible to many. Questions to be addressed in the text: How were bistable dynamics in these simulated networks with bifurcation parameter alpha identified (search space = product of weight matrices [w_ij] and alpha values?) How exhaustive was the search, etc..)? What was criterion for bistability? (You used BIC, but between what models? – one has to guess: it is for unimodal vs bimodal distribution ?)? Why does the bifurcation point change with N_sample (earlier in larger N_sample in Fig 1)? Etc...
Also: I am not familiar with bistability in high-dimensional systems. Specifically, the pitchfork bifurcation (eq 6) is not structurally stable, at least in the ODE form, not sure how this is in the Hopfield network form. Thus, an expansion of this last section that provides more detailed explanation of this model for the simulation would be useful.

We thank the reviewer for pointing out these aspects of the simulation model that could be easily clarified.

First, for the purposes of a simple test of the method, we chose weight matrices to be homogeneous and all-to-all. In this case the bifurcation point can be solved analytically in the limit of small noise, as we now say explicitly: "In the limit of small noise, the model has a transition to bistability at $\alpha\_crit = 1/N\_genes$, as can be shown analytically using mean-field theory". We now also add that "These bifurcations can also be found numerically in non-symmetric, heterogeneous cases, in which two parameters must be tuned (as a pitchfork bifurcation has codimension 2) [56], though we do not explore this more complicated case here." This is also related to the reviewer's point that the pitchfork bifurcation is not structurally stable. While the pitchfork does generally require two parameters to be simultaneously tuned, in this simple model the symmetry between ON and OFF states automatically constrains one of those two dimensions.

Second, we now explicitly mention in the Results section the models that we compare in the Bayesian analysis: "a Bayesian Information Criterion comparing the unimodal Gaussian distribution to the bimodal distribution expected near a transition strongly favors bimodality at these later ages (Fig. 2B; see Methods for more details)."

Finally, as we now point out in the Methods section, "Note that the bifurcation point does not change with increasing *N*_samples, only our ability to detect the bistability."

> *(5). Line 113: for this statement of the direction of the largest variance at bifurcation, for clarity please point to the text of lines 331+ or summarize the explanation here in main text.*

We have now added a reference to the relevant Methods section.

Reviewer #2

> *The authors present a method of detecting bi-stability in gene expression. They applies their method to a honey bee development dataset.*
>
> *The presentation, figures and method are nicely clear.*

We thank the reviewer for their time and useful feedback.

> *Nevertheless, I think this work should not be considered for publication, for the following (main) reason: it appears as an (incomplete) mixture of three different subjects, instead of a work with clearly defined contours. I suggest that the authors should focus more deeply in one of the following subjects instead mixing them together:*
>
> *1) An experimental work around development transition in honey bees, with the starting point as bi-stable genes. Among these identified genes, they role should be clarified: are they transition "drivers" or consequences of the transition? What happen at the single-*

tissue/single cell level? What happen if these genes are knock-down/over-expressed? Such a work should include other experimental data.

2) A bioinformatic work that propose a bi-stability identification method. The author method should be applied to many other datasets that are expected to have bi-stability, eg cell differentiation datasets. Is their method able to identify genes that are known to be bistable? How does their method depend on noise in dataset? Is it necessary to have different time points? Is their method efficient for all large dimension experimental data (bulk RNA-seq, sincgle-cell RNA-seq, quantitative proteomic data, etc.)?

3) A modeling work that is able to propose a mechanistic explanation when bi-stable expression is observed. For that, they should consider the different bistable distributions (Landau, mixed gaussian with equal variance, mixed gaussian with unequal variances, etc.). They also need to consider different type of modeling framework that is able to produce a bi-stability (ODE based of chemical dynamics, ODE based on Michaelis-Menten, agent based methods, Boolean approaches, etc.).

We view our work chiefly as a proof-of-concept that our approach can provide insight into the biology of a particular case of a critical transition. We focus on the honey bee transition because this is our expertise. Nonetheless, we agree with the reviewer that demonstrating our approach on another existing dataset that is expected to show bistability is relevant to show its generality and to clarify its relationship with other existing methods. We therefore demonstrate the use of our method on an example of hepatocellular carcinoma data, now detailed in the last paragraph of the section "Comparison to other potential methods for identifying a transition" and the new Figure 6. In particular, this shows that our method is able to handle much larger datasets, in this case one that measures the expression of tens of thousands of genes. As we point out in this new section, this analysis also demonstrates the relationship between our method and existing methods that look for increased fluctuations:

> While SNE looks for fluctuations indicative of a transition to bistability that is imminent but has not yet happened, our approach is designed to identify bistability as soon as possible after the transition has occurred. We therefore expect our method to identify bistability at the same stage or soon after the stage identified by SNE. When we apply our Landau approach to the data (Figure 6) we indeed find strong and growing evidence of bistability starting at the "early HCC" stage.

As for finding a "*different type of modeling framework that is able to produce a bi-stability*": This is an interesting point, though a thorough analysis is beyond the scope of the current manuscript. In fact, we expect that most of the approaches that the reviewer mentions will produce the same results regarding bistability. We now include this point in the Discussion:

> The assumptions we make in deriving the form of the bistable distribution are minimal: interaction networks must be dense enough for the mean-field Landau theory to be a good approximation, and dynamics must be sufficiently continuous so that variation along the principal component can be described well by a continuous distribution. These assumptions are likely to hold in many biological networks, making our approach

promising for interpreting the dynamics of gene expression in other organisms and perhaps even more generally across systems biology.

These assumptions are true for a variety of mechanistic models that the reviewer mentions, and we therefore find it unlikely that we would be able to distinguish one best mechanistic model given only the observation of bistability.

Additional comments:
- The choice of the list of genes is not clear (first paragraph of results). Are they directly extracted from references 29/30?

We now include a more thorough explanation of the gene selection process in the Methods section:

Genes were selected from the honey bee *Apis mellifera* assembly and gene annotation information at NCBI (https://www.ncbi.nlm.nih.gov). Genes were prioritized using a pragmatic approach similar to Refs. [47, 48]. In brief, our strategy integrated information on a specific biological process (the nurse-forager transition) with information on genes that were suggestively, correlatively, or potentially causally involved. Thus, gene selection relied on a process of extensive literature review as well as interdisciplinary biological expertise. The gene prioritization was reviewed by three members of the interdisciplinary team (YW, GVA, and REP) to minimize operator biases.

- Laudau distribution "seems" to fit better than mixed gaussian one; could the authors provide a test for it? What is the exact number of free parameters for both of these distributions (it seems that mixed gaussian has 3...)?

We now quantify the fit of each distribution using a KS test:

Comparing the best fit of the two distributions to 50,000 samples from the model close to the transition (Fig. 3B), the Landau distribution is not significantly different from the sampled data (KS statistic = 4.2 x 10^-3, p=0.33), while the Gaussian mixture distribution, with one more parameter than the Landau model, is significantly different (KS statistic = 0.017, p<10^-10).

The mixed Gaussian model has 4 parameters (the mean of each group, the variance, assumed to be equal for each group, and the relative weight of the two groups). Note that the Landau distribution has fewer parameters (3) than the Gaussian mixture (4) and still fits better, as we now point out explicitly.

Reviewer #3

Daniels BC et al propose a statistical method for identifying bistability near a continuous transition directly from high-dimensional gene expression data. They applied the method

to a transcriptomic dataset of 91 pre-selected genes from honey bees at different developmental time points, showing successful identification the emergence of a bistable gene expression pattern statistically visible at age 10 days and more apparent at age 15 days.

Although model proposed is very original and of interest to a large community, further analyses are needed to evaluate the results.

We thank the reviewer for their time and their appreciation of our results.

Major comments

1. The proposed approach could be useful for interpreting existing gene expression data, both in the context of social insects and in other biological contexts. Authors should provide details of the computational implementation of the method and possibly make it available for downloading and testing.

We have now released on GitHub all code needed to reproduce our analysis on our dataset and apply it to other datasets: https://github.com/Collective-Logic-Lab/landau

2. The criteria for having fixed the data set size at 91 genes are not discussed. Simulations should be performed to test whether the number of genes analyzed could affect the method's ability to identify the transition state.

Given the theoretical expectation that variance along the bistable dimension will always dominate, we need only to find the bistable dimension using PCA. In this way, the dimensionality reduction provided by PCA makes it possible to include a large number of genes without affecting our ability to find the transition. Measuring additional genes will either not change anything about the principal component (if they are uncorrelated with the remaining genes) or will add to the ability to discriminate the bistable states (if they are correlated with the transition).

We now demonstrate this explicitly by running simulations with 10 genes and 1000 genes, showing that the method performs similarly to the 100 gene case in successfully identifying the transition. In the new Figure 8, we show an analogous plot to Figure 1 that demonstrates the performance of the method in these cases.

We also now analyze an example dataset that includes tens of thousands of genes, as detailed in the last paragraph of the section "Comparison to other potential methods for identifying a transition" and the new Figure 6. We are successful in finding the transition state in this large dataset soon after the bistability appears, which we now explicitly mention in the Discussion: "In the hepatocellular carcinoma example (Figure 6), we show that the method is able to handle large datasets with tens of thousands of genes."

3. The authors state that the method can identify individual genes that are related to transition states through a measure of proportion of their variance s. It is not explained

and shown whether the genes with the highest s are also expected to be those with the highest BIC bimodality coefficient.

We derived the measure *s* with the intuition that genes with the largest proportion of their variance along the bistable dimension would carry the most information (at least the most information available by measuring the expression of single genes) about which of the two bistable states the system is in. The reviewer suggests here that a simple measure of this information would be the degree to which bimodality is visible when measuring a single gene. In our tests, however, the ΔBIC measure is not useful in ranking the contributions of individual genes because this measure will also pick out genes that, for instance, display two distinct clusters but with a small variance that does not contribute strongly to the collective-level principal component.

Instead, we now further explain our intuition about the *s* measure by comparing it to the correlation *c* between the expression of individual genes and the expression projected along the bistable dimension (the principal component $\hat{v}$). As we now detail in the Methods section and the new Figure 7:

> While we know, by construction, that genes more correlated with $\hat{v}$ will have more of their variance aligned with $\hat{v}$, the measure *c* is somewhat more intuitive in the sense that individual genes with large *c* by definition provide more information about where the system is along the bistable dimension. We demonstrate the close relationship between *s* and *c* in Figure 7.

> *4. The results and the relevance of the proposed model should be corroborated by the analysis of an independent transcriptomic dataset, such as the recently published dataset in (Zhang W et al, iScience 2022) publicly available at GEO NCBI : GSE184507 or other relevant datasets.*

Though the dataset mentioned here is relevant to our honey bee context, it is unfortunately not useful for locating a developmental transition at the level of individual bees: The study measures gene expression in a large number of single cells, but only in a small number of bees, and we expect our method to need at least ~10 sampled bees to work well. Instead, as mentioned above, we have demonstrated the use of our method on another transcriptomics dataset that tracks the development of hepatocellular carcinoma in humans. In this independent dataset, our method successfully finds a case of bimodality that has previously been identified using another method (SNE). As we now point out in the Results and in our response to another reviewer,

> The SNE approach found evidence for a pre-critical state in the "very early HCC" stage [18]. While SNE looks for fluctuations indicative of a transition to bistability that is imminent but has not yet happened, our approach is designed to identify bistability as soon as possible after the transition has occurred. We therefore expect our method to identify bistability at the same stage or soon after the stage identified by SNE. When we apply our Landau approach to the data (Figure 6), we indeed find strong and growing evidence of bistability starting at the "early HCC" stage.
