# Supplementary material for: Identifying a developmental transition in honey bees using gene expression data
Source: PLoS Comput Biol. 2023 Sep 21;19(9):e1010704. doi: 10.1371/journal.pcbi.1010704 (PMC10547183; doi:10.1371/journal.pcbi.1010704)
Supplement: Attachment — Submitted filename: bistability_round_two_reviewer_response_v2.pdf [file pcbi.1010704.s003.pdf]

We would like to thank the reviewers again for their feedback. Below, we respond in detail to all comments, with reviewer comments in *italics* and our changes to the manuscript highlighted in green.

Reviewer #1

> *I am satisfied with the authors' revisions - they have addressed all my specific concerns satisfactorily. Especially, of paramount importance are the three possibilities in point (1) regarding the levels of interactions, which is now discussed in sufficient detail.*
>
> *A minor point: I am not a statistician to be able to say with certainty that the statement in lines 159 ff ("Comparing the best fit ..") is correct. It sounds reasonable to me, but here the problem is that of a type II error (failing to reject the null hypothesis that the two curves being compared are the same, when it is FALSE) - The author seem to just use KS and p-values in the Type I error setting. The KS test tends to have very low p-values which sets the bar high - which is good. But is good? It should be a matter of statistical power (what should beta value be?) and presented as such instead of Type I error situation (statistical significance of difference). Just my 2 cents on this point... Some additional verbiage of clarification may help.*

The reviewer brings up a valid point that our analysis with a fixed number of samples also has a fixed power: that is, if we were to use more samples, at some point any small differences between the distributions would be detectable and the KS test would reject the null hypothesis that the two are exactly the same. Effectively, the number of samples sets the power of the test.

Our goal here is to demonstrate that our analytical distribution is a good approximation to the simulated data. The KS statistic is a measure of this difference between distributions, and we include the p-value for a Type I error merely to demonstrate that this magnitude of difference is not unexpected given the number of samples. We now clarify this in the text, changing the description of our tests:

> Comparing the best fit of the two distributions to 50,000 samples from the model close to the transition (Fig. 5B), the Landau distribution is a notably better approximation to the sampled data than the Gaussian mixture model. Even for this large number of samples, the Landau distribution is not statistically distinguishable from the sampled data (KS statistic = 4.2 x 10^-3, p=0.33), while the Gaussian mixture distribution, with one more parameter than the Landau model, is significantly different (KS statistic = 0.017, p<10^-10).

To the reviewer's suggestion of a power analysis in terms of Type II errors: This would be appropriate if we wanted to be sure that we would be able to determine whether our samples were consistent with some second distribution that had a small, predetermined distance ("effect size") away from the Landau distribution. But given that there is no simple way to measure effect size in this context, we feel that this is not a particularly illuminating way to describe our results.

Reviewer #2

> *The author took into account my comment. They answered to all of them and modify the text accordingly.*
>
> *Nevertheless, I find that the modification are quite minimal, their work still appear as an incomplete mixture:*
>
> *- They applied their method to another example, but they didn't explain for what type of (omics) data their method can be used (and for which type of data their method cannot be used)*

We agree that it is an interesting question to consider whether our analysis might be general enough to be used across other types of data beyond gene expression. Previously, we limited our discussion of potential data types to gene expression and neural activity, cases for which we can more easily interpret the results in terms of collective decisions.

To emphasize the potential to analyze other types of data and to more clearly specify the requirements for the method to work, we added the following paragraph to the Discussion:

> More generally, the method could be useful whenever there is variability among individual samples that may undergo a continuous transition into separate types, and it is particularly valuable for interpreting high-dimensional datasets. Any type of -omics data could be used for the analysis, given two basic requirements. First, there must be sufficient separation of timescales that an equilibrium approximation is sensible, with fast dynamics that are roughly equilibrated over the slower timescale of the transition to bistability. Second, the data must include a sufficient number of sampled individuals at each timepoint (a minimum of roughly 10) in order for the bistable Landau distribution to be statistically distinguishable from a simpler Gaussian distribution.

> *- They assumptions are "likely to hold in many biological network". This is not very precise: what are the exact mathematical definition of their assumptions? For what class of dynamics are their method applicable (and for what class of dynamics are their method not applicable).*

The reviewer makes a good point that we were less than clear about situations in which we expect our method to fail. We now unpack our assumptions more explicitly in the Discussion. The precise mathematical definitions of these assumptions are found in the Methods sections 5.3 and 5.4. Note that we view our work as a proof of concept that the method can work across multiple systems, and we have not yet rigorously tested whether the method indeed fails to detect transitions in these cases.

In particular, the method is designed to detect continuous transitions that conform to Landau theory, so there may be other types of transitions (e.g. discontinuous, or continuous with a different shape) that would not be readily detected. We now include a new paragraph in the

Discussion that details conditions under which a distribution more complicated than our simplified Landau case could be a better approximation:

> If our method fails to detect a transition, this indicates either that there is no transition to bistability or that there is a transition of a type that cannot be well-described by the Landau distribution. Further work will be necessary to distinguish possible cases that could evade detection in this way. As a start, we highlight three assumptions in our derivation (set out more precisely in sections 5.3 and 5.4) that may not be met by some biological systems and therefore may lead to transitions that would not be well-described by our method. First, we assume a symmetric transition, with no bias toward either of the bistable types. Such a bias could be better represented by a more complicated distribution, but would require more parameters and therefore more data to reliably infer. Second, our derivation assumes continuous time and continuous variables, whereas some regulatory systems (with, for instance, bursty dynamics or highly nonlinear saturating functions) may not easily conform to this. Finally, we assume that the interaction network is sufficiently dense for a Landau-type mean-field theory to be a good approximation. In spatially organized low-dimensional systems studied in statistical physics, correlated fluctuations are known to cause mean-field models to become invalid [Pathria 2011], such that the expansion in Eqs. (5) and (6) is not justified. This could potentially lead to a transition with a different form than we derive. On the other hand, mean-field theory is known to work well even in relatively small spatial dimensions (e.g. 4 or larger for the well-known Ising model), when connectivity becomes sufficiently dense. Due to the prevalence of feedback and relatively dense connectivity of biological networks, we are optimistic that mean-field theory represents a good approximation.
>
> *That is why I do not include a recommendation.*

Reviewer #3

> *The authors have responded comprehensively to all raised points, giving special attention to key aspects such as the method's robustness concerning the number of measured genes. They have also provided a more elucidating explanation of the "s" measure by drawing a comparison to the "c" correlation between the expression of individual genes and the expression projected along the*
> *bistable dimension. Additionally, the authors have showcased the efficacy of their method on an independent transcriptomics dataset, further validating its performance and applicability.*

We thank the reviewer again for their time and their appreciation of our results.
